# Analysis of hollow wall effect on the fluid dynamics in the orbitally shaken bioreactors

**DOI:** 10.1038/s41598-022-13441-5

**Published:** 2022-06-10

**Authors:** Likuan Zhu, Weiqing Chen, Chunyang Zhao

**Affiliations:** Shenzhen Key Laboratory of High Performance Nontraditional Manufacturing, College of Mechatronics and Control Engineering, Shenzhen University, Shenzhen, 518060 China

**Keywords:** Animal biotechnology, Biomedical engineering

## Abstract

Orbitally shaking bioreactors (OSRs) have recently been increasingly applied in the biopharmaceutical industry because they can provide a suitable environment for mammalian cell growth and protein expression. Fluid dynamics information is crucial for analyzing or optimizing of different types of bioreactors. Considering that the structure has an important influence on the fluid dynamics in a bioreactor, it necessary to design or optimize its structure by the computational fluid dynamics (CFD) approach. The aim of this study is to optimize the wall structure of a hollow cylinder OSR proposed in our previous work. Based on previous research, the influences of the hollow wall of the OSR on fluid dynamics and the volumetric mass transfer coefficient ($k_{L}a$) were analysed by the established CFD model. The results showed that the mixing performance of OSR could be improved by decreasing the installation height of the hollow wall. An installation height of 30 mm was found to be most favourable for mixing. The reliability of the CFD model was verified by comparing the liquid wave height and liquid wave shape between the simulation and experiment. The shear stress in the hollow cylinder OSR was proven gentle for mammalian cell cultivation.

## Introduction

Bioreactors are critical equipment used for mammalian cell culture. Currently, stirred tank bioreactors (STRs) and orbital shaken bioreactors (OSRs) are common types of bioreactors widely used in either laboratory or pilot-scale mammalian cell cultivations^1–3^. In recent years, OSRs have become increasingly popular because of their simple stirring principle, low cost, simple operation and suitability for disposable experiments^4,5^. Moreover, the orbital shaking motion of OSRs could prevents sedimentation and enhance gas exchange, avoiding the damagingly high shear rates relative to STRs^6^. As an important disposable bioreactor, improving the mixing performance of OSRs is necessary. Research has shown that different structures of OSRs have different effects on mixing performance^7,8^. For example, introducing a vertical baffle on a wall is an effective way to improve turbulence characteristics and mixing performance^9^. A helical track was proven valid for increasing viable cell density in suspension cultivation^10^. A vaulted “bump” was proposed on the bottom wall, and the result showed that the mass transfer rate was enhanced significantly and that cell accumulation near the centre of the bottom wall could be avoided, which is preferable for suspension cultivation with a high viable cell density^11^.

Computational fluid dynamics (CFD) simulation is a reliable numerical analysis technology^12,13^. Compared to traditional experimental techniques, CFD simulation can save capital and labour and be used in many different situations^14,15^. Considering that it can provide a deeper understanding of bioreactor fluid dynamics and reduce the number of models, CFD simulation has been seen as a valuable tool for analysing bioreactors, such as STRs and OSRs^16–18^.

In a previous study^19^, we proposed a new type of OSR with a hollow cylindrical wall. For this kind of OSR, the ratio of the outer cylinder diameter to the intercylinder diameter is the key structural parameter. In that prototype study, the value d$_{i}$/d was optimized, and a suitable value of 0.4 was suggested18. However, the mass transfer capability was still low in some specific regions, which strongly indicated that the structure of the hollow cylindrical wall could be further optimized. Thus, the aim of this study is to continue focusing on the OSR with a hollow cylindrical wall and analysing the effect of the installation height of the hollow structure on the mixing performance, volumetric mass transfer coefficient ($k_{L}a$) and shear stress in the OSR by the CFD method.

## Materials and methods

### Bioreactor

**Figure 1:**
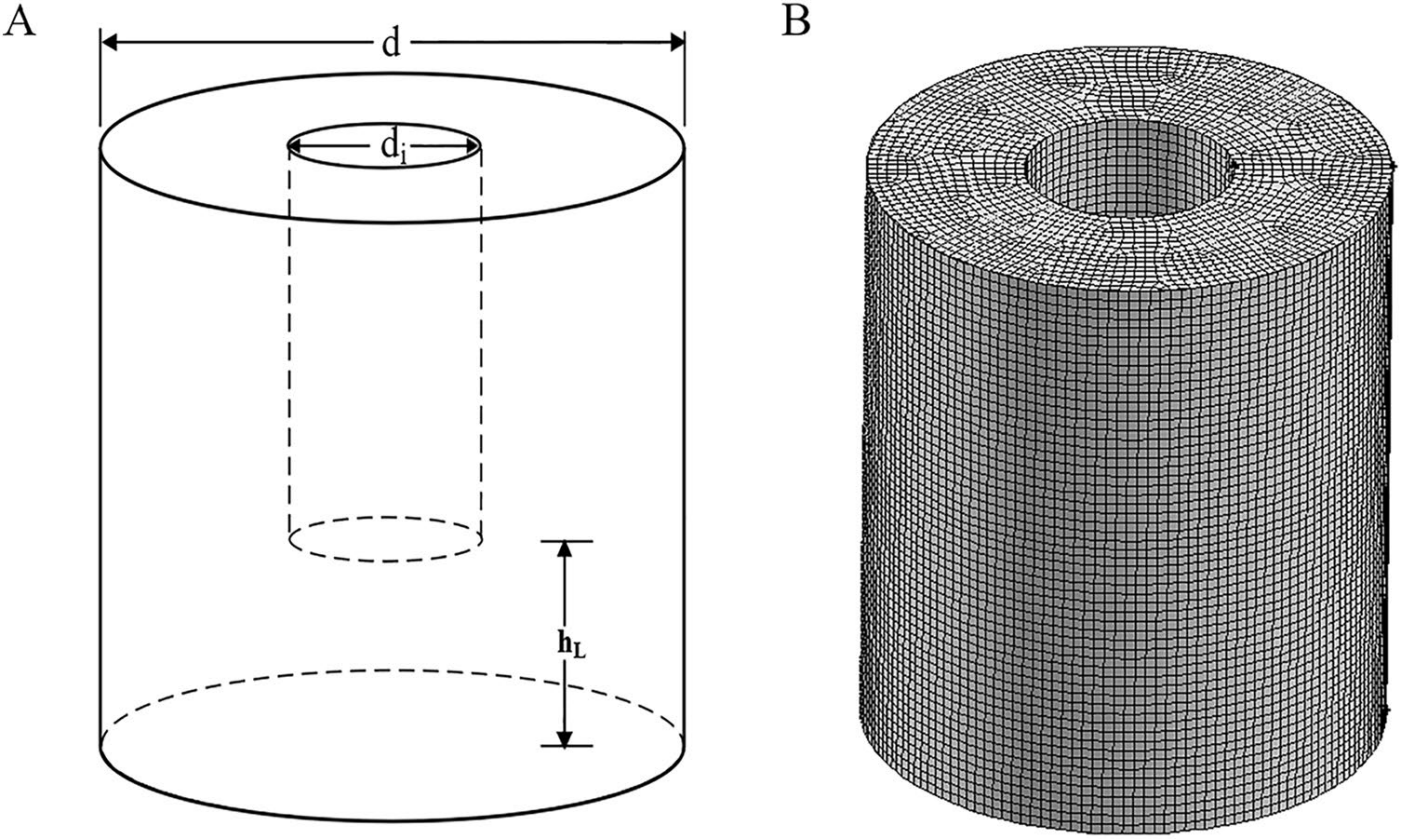
The geometry (**A**) and mesh structure (**B**) of the hollow OSR with d$_{i}$/d of 0.4. This geometry had several important geometric parameters, d was the diameter of the outer cylinder wall, d$_{i}$ was the diameter of the inner cylinder wall, and h$_{\mathrm {L}}$ was the installation height of the inner cylinder from the bottom. The geometric volume was about 24 L, and the maximum working volume was about 10 L. The mesh number was $\mathrm {3.4\times 10^{6}}$ for the hollow OSR.

The OSR with a hollow cylindrical wall was orbitally shaken on an ES-X shaker (Kühner AG, Biersfelden, Switzerland) with a shaking diameter of 50 mm. In this study, the total volume of the hollow cylindrical OSRs was approximately 24 L, the height of the vessel was 0.35 m, the outer diameter (d) was 0.3 m, and the inner diameter (d$_{i}$) was 0.12 m. All experiments and simulations in this paper were carried out under the conditions of a filling volume of 8 L and shaking speed of 100 rpm. The installation height of the intercylinder wall from the bottom was represented by h$_{\mathrm {L}}$ (see Fig. 1).

### Liquid wave capturing

The moving gas-liquid interface was captured by a camera from an Apple Mobile Phone (iPhone 12, Apple Inc., California, USA) at a slow motion model with 240 fps. The phone was fixed on the shaking platform to keep it relatively stationary with the hollow OSRs. To see the liquid more clearly, a certain amount of Methyl Red (1 M/L) and hydrochloric acid (1 M/L) was added before performing the capturing experiment.

### CFD model

Reynolds mean mass and momentum conservation equations are used to control fluid flow (Eqs. (1), (2) and (3)):

1 $$\begin{aligned} \frac{\partial \rho }{\partial t}+\triangledown \left( \rho V \right)= & {} 0 \end{aligned}$$

2 $$\begin{aligned} \frac{\partial }{\partial t}\left( \rho V \right) +\triangledown \left( \rho VV \right)= & {} -\triangledown p+\triangledown \left( \tau \right) +\rho \vec {g}+\vec {F} \end{aligned}$$

3 $$\begin{aligned} \triangledown= & {} \frac{\partial }{\partial x} +\frac{\partial }{\partial y}+\frac{\partial }{\partial z} \end{aligned}$$

where $\rho$ refers to the density of the fluid ($\mathrm {kg \,  m^{-3}}$), *p* refers to the pressure (Pa), $\tau$ refers to the shear stress (Pa), $\rho \vec {g}$ and $\vec {F}$ refer to gravity and the external force, respectively, and *V* represents the linear velocity vector.

The shear stress can be obtained from Eq. (4):

4 $$\begin{aligned} \tau =\sqrt{\tau _{xy}^{2}+\tau _{yz}^{2} +\tau _{xz}^{2}} \end{aligned}$$

where $\tau _{xy}$, $\tau _{yz}$, $\tau _{xz}$ represent the three directions of shear stress.

The three shear components are determined as follows (Eqs. (5), (6) and (7)):

5 $$\begin{aligned} \tau _{xy}= & {} \mu \left( \frac{\partial V_{x}}{\partial y}+\frac{\partial V_{y}}{\partial x} \right) \end{aligned}$$

6 $$\begin{aligned} \tau _{yz}= & {} \mu \left( \frac{\partial V_{y}}{\partial z}+\frac{\partial V_{z}}{\partial y} \right) \end{aligned}$$

7 $$\begin{aligned} \tau _{xz}= & {} \mu \left( \frac{\partial V_{x}}{\partial z}+\frac{\partial V_{z}}{\partial x} \right) \end{aligned}$$

where $\mu$ is the dynamic viscosity.

### The volumetric mass transfer coefficient model

The volumetric mass transfer coefficient ($k_{L}a$) is crucial for the bioreactor, which analyzes key parameter of the oxygen transfer rate. The computed value of $k_{L}a$ was obtained by simulating the mass transfer coefficient ( $k_{L}$) and specific interface area (*a*) respectively.

The specific interface area (*a*) can be obtained from Eq. (8):

8 $$\begin{aligned} a=\frac{A}{V_{L}} \end{aligned}$$

where the *A* is the interface area (m^2^) which could be obtained by the volume of fraction (VOF) model (see Table 1), and the $V_{L}$ is the filling volume (L).

The mass transfer coefficient ($k_{L}$) can be obtained from Eq. (9):

9 $$\begin{aligned} k_{L}=K\cdot \sqrt{D_{L}}\cdot \left( \frac{\varepsilon }{\upsilon } \right) ^{\frac{1}{4}} \end{aligned}$$

where $K=$ 0.4 is the model constant, $D_{L}$ is the diffusion coefficient of oxygen in the water $\left( \mathrm {m^{2} \,  h^{-1}}\right)$, $\varepsilon$ is the energy dissipation rate $\left( \mathrm {m^{2} \,  s^{-1}}\right)$ and $\upsilon$ is the kinematic viscosity of water $\left( \mathrm {m^{2} \,  s^{-1}}\right)$.

**Table 1:** Simulated wave pattern properties of orbitally shaken bioreactors (OSRs) at different installation height.

Installation height (mm)	Wave height$_{max}$ (mm)	Wave height$_{min}$ (mm)	*A* (mm)	$\Delta h$ (mm)	$\varepsilon$ $\left( \mathrm {m^{2} \, s^{-3}}\right)$
0	198.400	60.140	0.071	138.260	0.038
30	197.710	57.720	0.070	139.990	0.036
60	197.280	57.010	0.070	140.270	0.037
90	196.210	56.560	0.073	139.650	0.036
120	200.710	59.160	0.081	141.550	0.035
Without baffle	200.60	64.240	0.080	136.360	0.027

The filling volume was 8 L and shaking speed was 100 rpm. The *A* was the value of interface area. The $\Delta h$ was the difference between the Wave height $_{max}$ and the Wave height $_{min}$. The $\varepsilon$ was the energy dissipation rate.

### Orbitally shaking movement

The orbital shaking motion naturally induces a homogeneous, rotating centrifugal force on the fluid^20^. This centrifugal force is considered by adding the source term to the Navier-Stokes equations in the CFD model. The centrifugal forces in Eqs. (10) and (11) are given as follows:

10 $$\begin{aligned} F_{x}= & {} \omega ^{2}R_{s}\cdot \mathrm {cos}\left( \omega t \right) \end{aligned}$$

11 $$\begin{aligned} F_{y}= & {} \omega ^{2}R_{s}\cdot \mathrm {sin}\left( \omega t \right) \end{aligned}$$

where $F_{x}$ and $F_{y}$ represent the centrifugal force $\left( \mathrm {m \,  s^{-2}} \right)$ in the x and y directions, respectively. $R_{s}$ represents the radius of shaking (m), and $\omega$ represents the angular velocity of shaking $\left( \mathrm {rad \,  s^{-1}} \right)$.

### Simulation conditions

All simulations in this paper were performed in ANSYS FLUENT 16.0 (ANSYS Inc., Canonsburg, PA, USA). In this paper, the volume of fluid (VOF) model was used to obtain the gas-liquid interface. The VOF model was employed to track the moving gas-liquid interface^21^. The $\mathrm {k-\omega -SST}$ turbulence model was used to enclose the governing equations of the fluid motion^22^. All the boundary conditions were set to the wall. The PISO algorithm was used to solve the velocity and pressure. The time step size was 0.0001 seconds. The maximum courant number was 0.25. The grid for this hollow OSR was generated using Gambit 2.4.6 (ANSYS Inc., Canonsburg, PA, USA).

## Results and discussion

### Model validation

**Figure 2:**
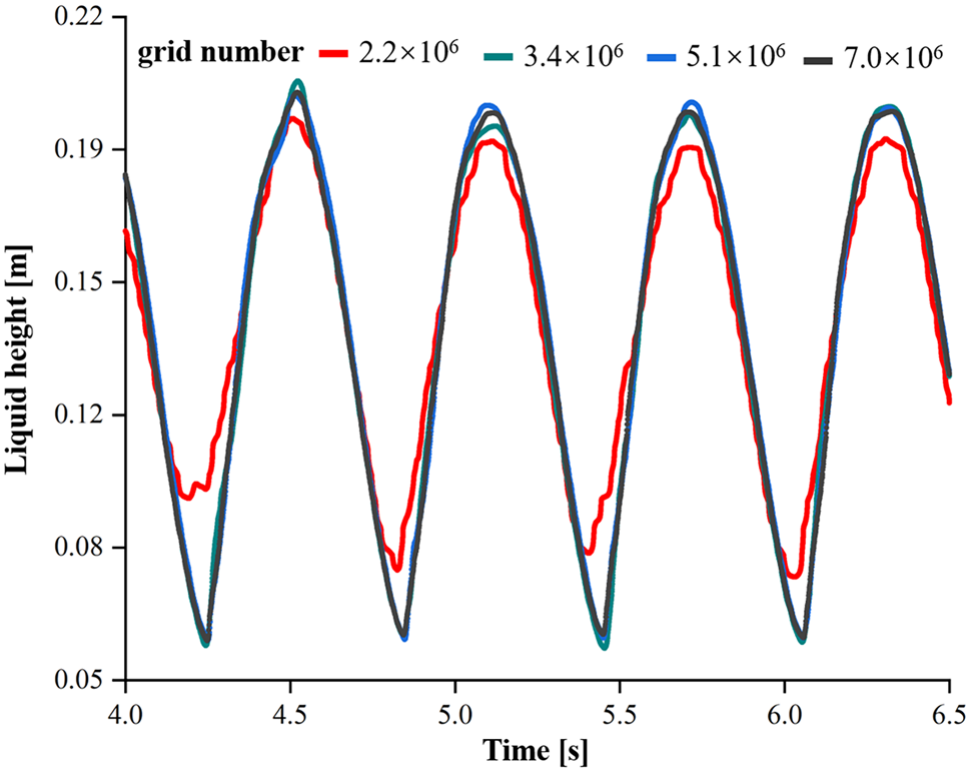
The hollow OSRs of the h$_{\mathrm {L}}$  = 0 mm with filling volume of 8 L and shaking speed of 100 rpm was simulated. The simulation of the hollow OSR used the mesh numbers of $\mathrm {2.2\times 10^{6}}$, $\mathrm {3.4\times 10^{6}}$, $\mathrm {5.1\times 10^{6}}$ and $\mathrm {7.0\times 10^{6}}$ to verify the independence of the mesh numbers. In order to obtain the shaking of liquid height with time, the liquid height was automatically saved every 30 time-steps (0.0001 s) during the simulation process. The height of the liquid at the interface was measured at the point of intersection with the fixed vertical line on the vessel wall.

To test the influence of the mesh number on the simulated result, four different mesh numbers of $\mathrm {2.2\times 10^{6}}$, $\mathrm {3.4\times 10^{6}}$, $\mathrm {5.1\times 10^{6}}$ and $\mathrm {7.0\times 10^{6}}$ were used to calculate the liquid height for the hollow OSR with an h$\mathrm {_{L}}$ of 0 mm. As the results show (Fig. 2), the liquid height curve would almost not change when the mesh number exceeded $\mathrm {3.4\times 10^{6}}$, which indicated that this mesh number ($\mathrm {3.4\times 10^{6}}$) was already sufficient to obtain stable and trustable simulation results. Thus, a mesh number of $\mathrm {3.4\times 10^{6}}$ was used for all the simulations in this article.

**Figure 3:**
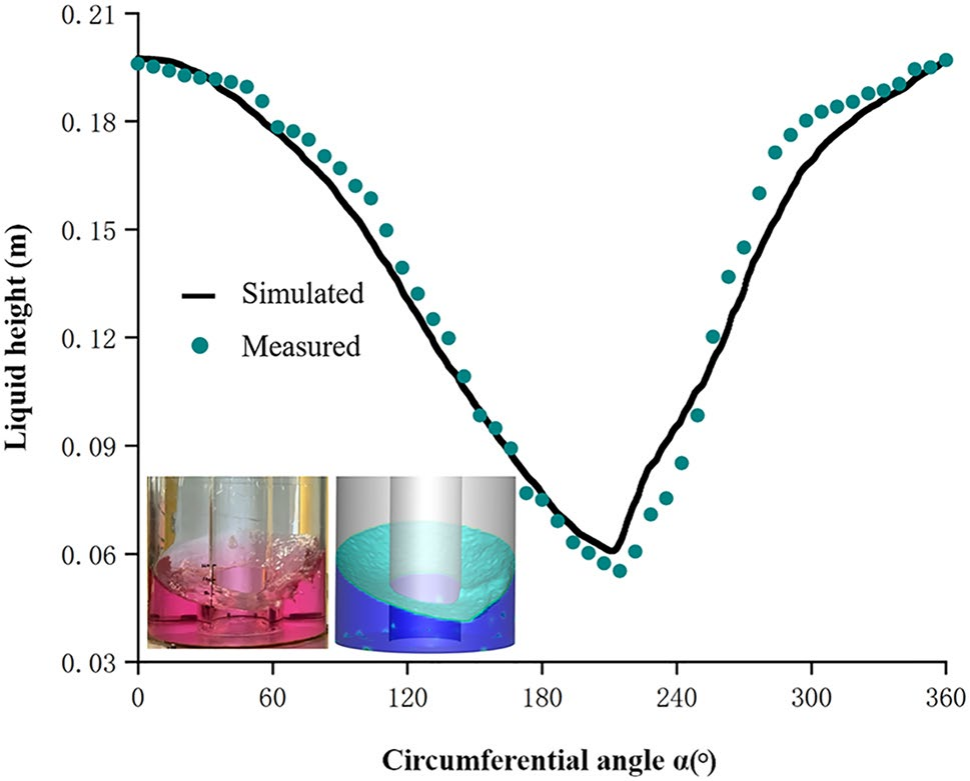
The liquid level of simulation and experiment was compared. The simulation and experimental results of the hollow OSR with filling volume of 8 L and shaking speed of 100 rpm under h$_{\mathrm {L}}$ = 0 mm were compared. To see the liquid more clearly, a certain amount of Methyl Red and hydrochloric acid was added before performing the capturing experiment. The solid black line and the green point represent the liquid wave height measured by simulation and experiment respectively. The circular angle ($\alpha$) represented the angle of the wall of the hollow OSR in the cylindrical coordinate system.

To validate the established CFD model, the calculated liquid wave height was compared with its measured values by a wave capturing experiment. As shown in Fig. 3, the simulated and measured liquid wave shapes were similar. However, the simulated gas-liquid interface was found to be smoother than the experimental observation. This drawback might be decreased by adopting a higher-order turbulence but with a penalty of a longer simulation time. By comparing the curves of the liquid wave in the simulated and measured results, the wave height difference was found to be less than 15$\%$, which indicated that the established CFD model was acceptable for use in analysing the fluid dynamics of OSRs with hollow structures.

### Flow field

**Figure 4:**
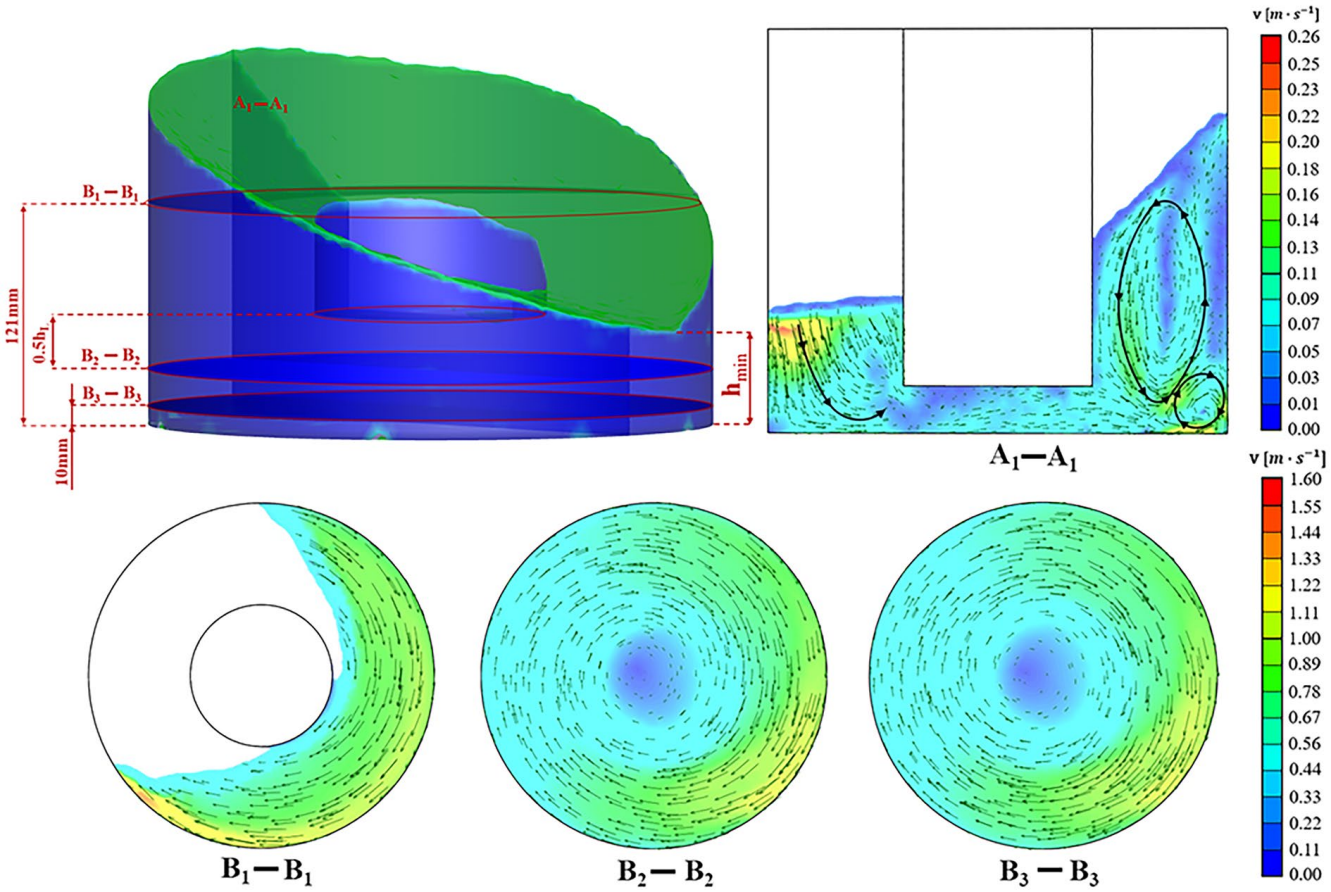
The velocity distribution of the flow field at different sections of the hollow cylindrical OSR with h$_{\mathrm {L}}$ = 30 mm. The fluid velocity vectors was calculated in a vertical interface (A$_{1}$-A$_{1}$) and three different horizontal sections (B$_{1}$-B$_{1}$, B$_{2}$-B$_{2}$ and B$_{3}$-B$_{3}$) respectively. The position of the vertical section was a plane of symmetry of the hollow cylinder. The heights of the three horizontal sections were 121 mm, 0.5h$_{\mathrm {L}}$ (15 mm) and 10 mm respectively. The color bar on the right showed the magnitude of the fluid velocity.

The fluid velocity is the basic information for the flow field of a bioreactor, and it determines whether other derived fluid parameters relevant to cell cultivation are correct. To generally understand the characteristics of this type of bioreactor, the fluid velocity distribution of the cylindrical OSR with an inner hollow wall for the installation height of 30 mm (h$_{\mathrm {L}}$ = 30 mm) is shown in Fig. 4. The maximum fluid velocity was found to be approximately 1.6 $\mathrm {m \,  s^{-1}}$ at the wave front near the vessel wall, which was slightly higher than its theoretical maximum value of 1.57 $\mathrm {m \,  s^{-1}}$ from Eq. (12)^2^, which may be caused by local turbulence^23^.

12 $$\begin{aligned} V_{max}=2\pi rN \end{aligned}$$

where $V_{max}$ represents the theoretical maximum fluid velocity, *r* represents the radius of the hollow cylindrical OSRs, and *N* represents the shaking speed.

Three vortices could be observed from the vertical section (A$_{1}$-A$_{1}$) in Fig. 4. The bulk fluid would be driven from the bottom of the bioreactor to the top along those vortices. A vortex was located on the left side of the vertical section and was in an underdeveloped state. This might be because the amount of fluid was not sufficient on this side. There is another subtle reason for this phenomenon, which was that the wave front was located at the left side with the maximum fluid velocity to transfer the mixing energy to other fluid particles. Therefore, it is reasonable that only a limited amount of fluid particles can follow the wave front closely, which causes the fluid volume to be smaller near the wave front. Two vortices were located at the wave crest side (right side). The larger one could drive fluid flowing along a larger circle (bottom to top) and was crucial for global fluid mixing in OSRs. For the smaller vortex, it could increase the mixing intensity at the corner of the bioreactor where mixing is not good and even the “velocity dead zone” occurs easily. Therefore, the existence of a smaller vortex was favourable for increasing the local mixing efficiency, which might explain why the velocity at the side corner of the wave trough is lower than that at the side corner of the wave crest. It can be observed that the maximum velocity is near the wall of the hollow OSRs, and the fluid near the vessel wall has a high velocity because of the high Froude numbers ($F_{r}=V^{2}/\left( gl_{0} \right)$, where *V* represents fluid velocity, *g* represents gravity acceleration, and $l_{0}$ represents characteristic length)^24^. The Froude number is the key dimensionless driving parameter, which represents the driving capability^25^. The maximum velocity was located on the wall side of the wave trough rather than the wave crest side. The mass transfer between the right and left vortices was also observed by the moving fluid in the middle of the bioreactor bottom. As Fig. 4A$_{1}$–A$_{1}$ shows, the percentage of fluid volume with different range velocities and fluid moving orientations are important for the energy exchange process.

To analyse the mixing properties in horizontal planes, the fluid velocity distributions were calculated on three different horizontal planes with different heights. As shown in Fig. 4B$_{1}$-B$_{1}$, B$_{2}$-B$_{2}$ and B$_{3}$-B$_{3}$, there was only one large vortex on the horizontal plane, and the vortex centre was almost identical to the plane centre.The high fluid velocity was found near the vessel wall due to the high wall velocity, and the low fluid velocity was located at the vortex centre.It should be noted that the maximum fluid velocity also occurred at the wave front, as mentioned before.

### Effect of the hollow vessel wall on the flow field

**Figure 5:**
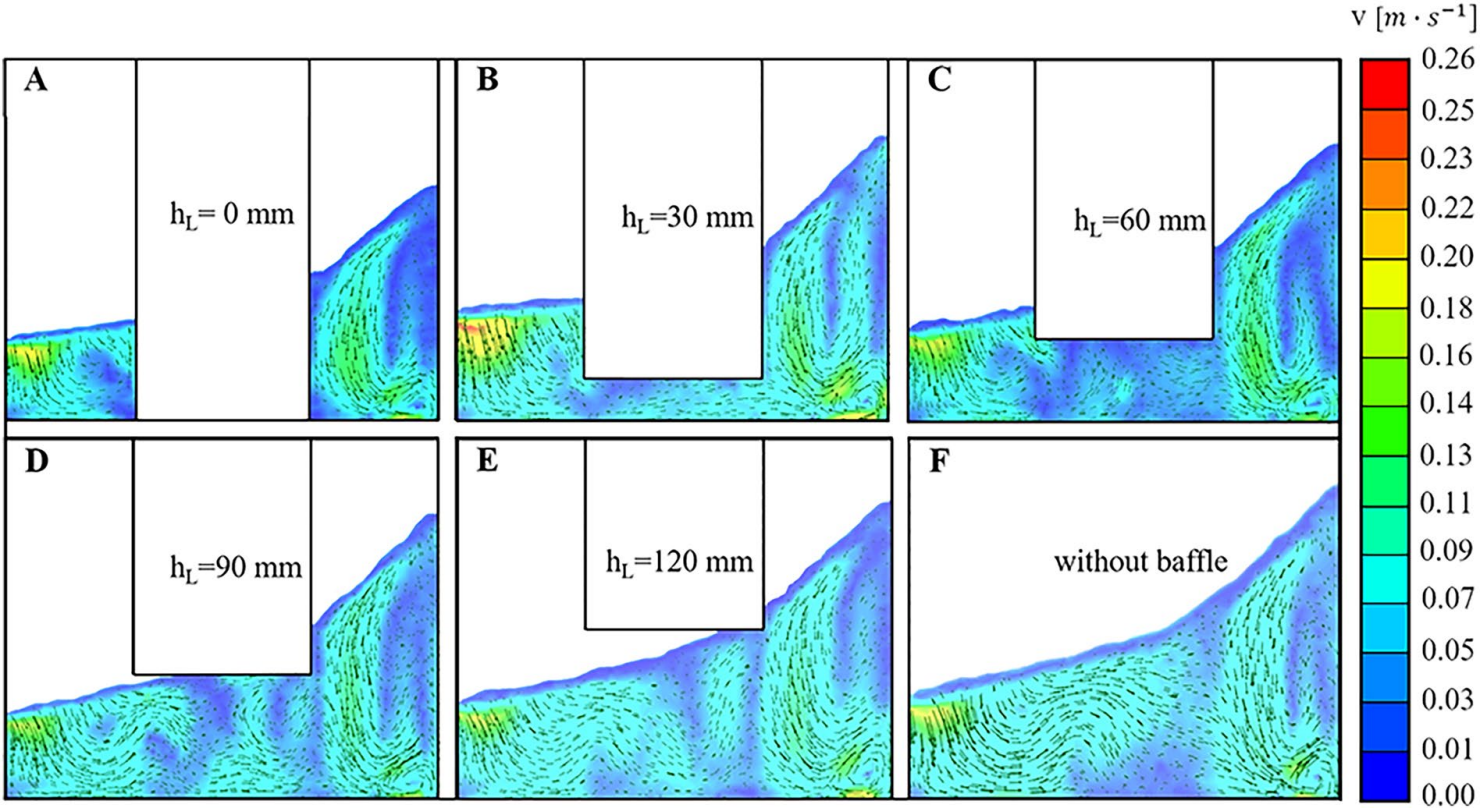
The fluid velocity distribution on vertical section of hollow cylinder OSRs with different installation heights. The position of the vertical section was a plane of symmetry of the hollow cylinder. The color bar on the right showed the magnitude of the fluid velocity.

To analyse the effect of hollow vessel wall on the fluid dynamics of OSRs, simulations were conducted on several cylindrical hollow OSRs with different installation heights with shaking speed of 100 rpm and filling volume of 8 L. In detail, the hollow vessel wall was installed at different positions with h$_{\mathrm {L}}$ = 0 mm (Fig. 5A), h$_{\mathrm {L}}$ = 30 mm (Fig. 5B), h$_{\mathrm {L}}$ = 60 mm (Fig. 5C), h$_{\mathrm {L}}$ = 90 mm (Fig. 5D), and h$_{\mathrm {L}}$ = 120 mm (Fig. 5E) away from the vessel bottom.

**Figure 6:**
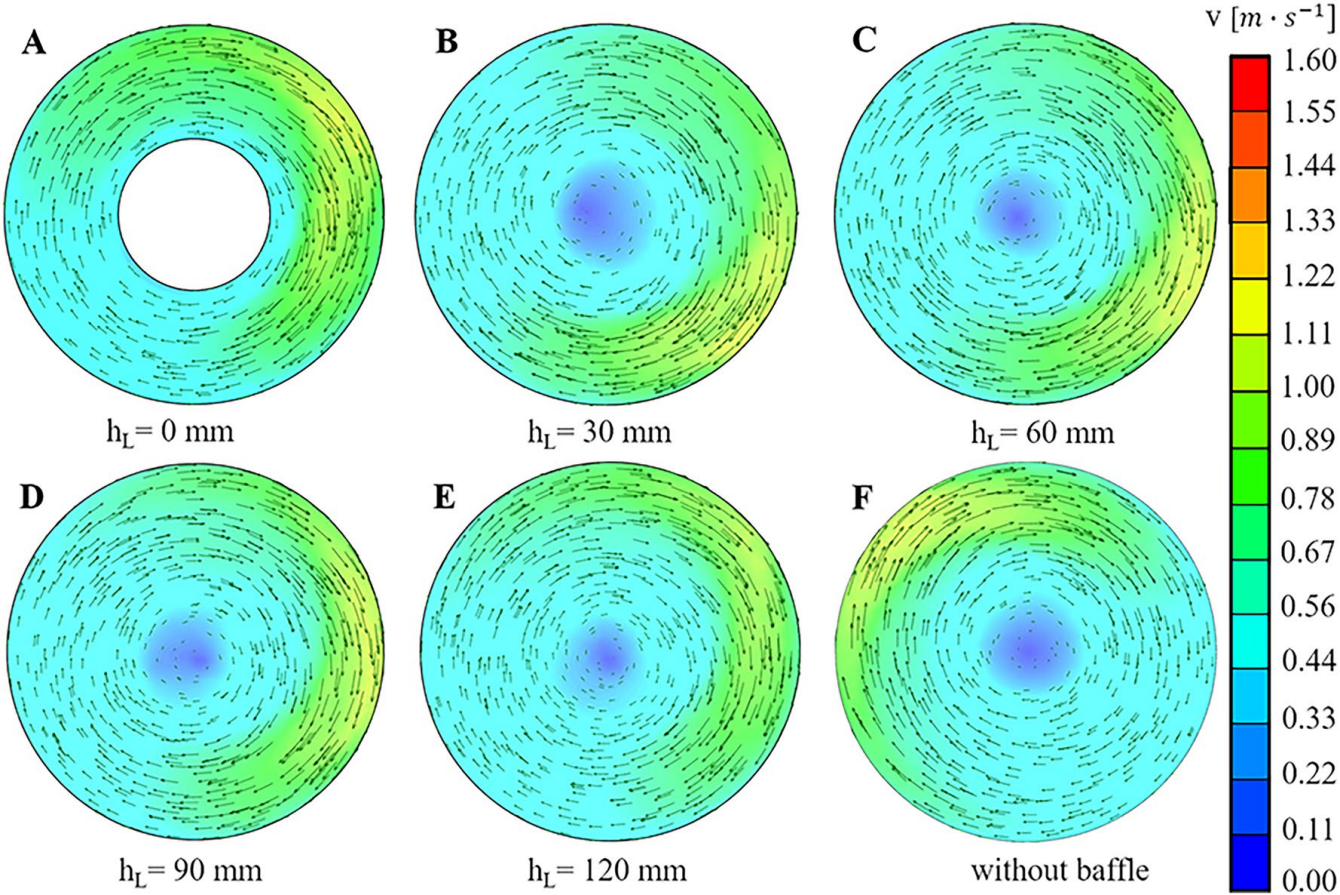
The fluid velocity distribution in fixed horizontal plane of hollow cylinder OSRs with different installation heights. The height between the fixed horizontal plane and the bottom wall of hollow cylinder OSRs was 10 mm.The color bar on the right showed the magnitude of the fluid velocity.

In Fig. 5, it can be observed that there are three vortices in each vertical section. Similarly, a vortex lies on the side of the wave trough side (the left side of the vertical section), which has not yet fully formed. The other two vortices were located on the wave crest side (the right side of the vertical section). The maximum velocity can be observed at the wall of the trough for each case. For all the maximum velocities, the maximum value of 0.26 m/s was found at the hollow OSR with h$_{\mathrm {L}}$ = 30 mm. In fact, the magnitude of the bulk fluid velocity was also higher in the hollow OSR with h$_{\mathrm {L}}$ = 30 mm (Fig. 5B) than in other cases. For the installation height of h$_{\mathrm {L}}$ = 120 mm (Fig. 5E), the flow field did not seem to change compared with the OSR without the hollow wall structure. The reason might be that there was only a small part of the hollow cylinder contacting the moving fluid in the OSR with h$_{\mathrm {L}}$ = 120 mm, which indicated that the hollow structure has almost no influence on fluid moving with 100 rpm shaking speed and 8 L filling volume. With decreasing h$_{\mathrm {L}}$, the contact area between the hollow structure and the moving fluid increased, and the fluid field started to change. When h$_{\mathrm {L}}$ decreased from 120 mm (Fig. 5E) to 60 mm (Fig. 5C), the fluid velocity in the vertical centre decreased gradually, which was caused by the hollow bottom suppressing the fluid motion. This kind of resistance effect could inhibit the energy exchange process from the left vortex to the right vortex and was unfavourable for bulk mass transfer. However, as h$_{\mathrm {L}}$ decreased from 60 mm (Fig. 5C) down to 30 mm (Fig. 5B), the fluid velocity suddenly increased at the bioreactor centre part, and the region worked like a “mass transfer tube” with a high speed, which was helpful for uniform mixing in the OSR. However, when the value of h$_{\mathrm {L}}$ decreased to zero, the mass exchange among those vortices at two sides was completely cut off, and some quite low fluid velocity region occurred near the hollow bottom, which probably meant that the nutrient supply would be insufficient at the local region easily. For the vortices at the two sides, the magnitude of the fluid velocity increased steadily as h$_{\mathrm {L}}$ decreased from h$_{\mathrm {L}}$ = 120 mm to h$_{\mathrm {L}}$ = 30 mm, which might be caused by the hollow structure decreasing pushing more fluid into a high velocity state. However, the exchange among those vortices was completely cut off when the value of h$_{\mathrm {L}}$ decreased to zero, which caused the low fluid velocity near the hollow structure. Fig. 6 shows that the fluid velocity distributions were similar. There was a large radial vortex on each section, and the maximum velocity was located at the wall, but the low fluid velocity occurred at the middle region near the vortex centre.

### Effect of the hollow vessel wall effect on the volumetric mass transfer coefficient

**Figure 7:**
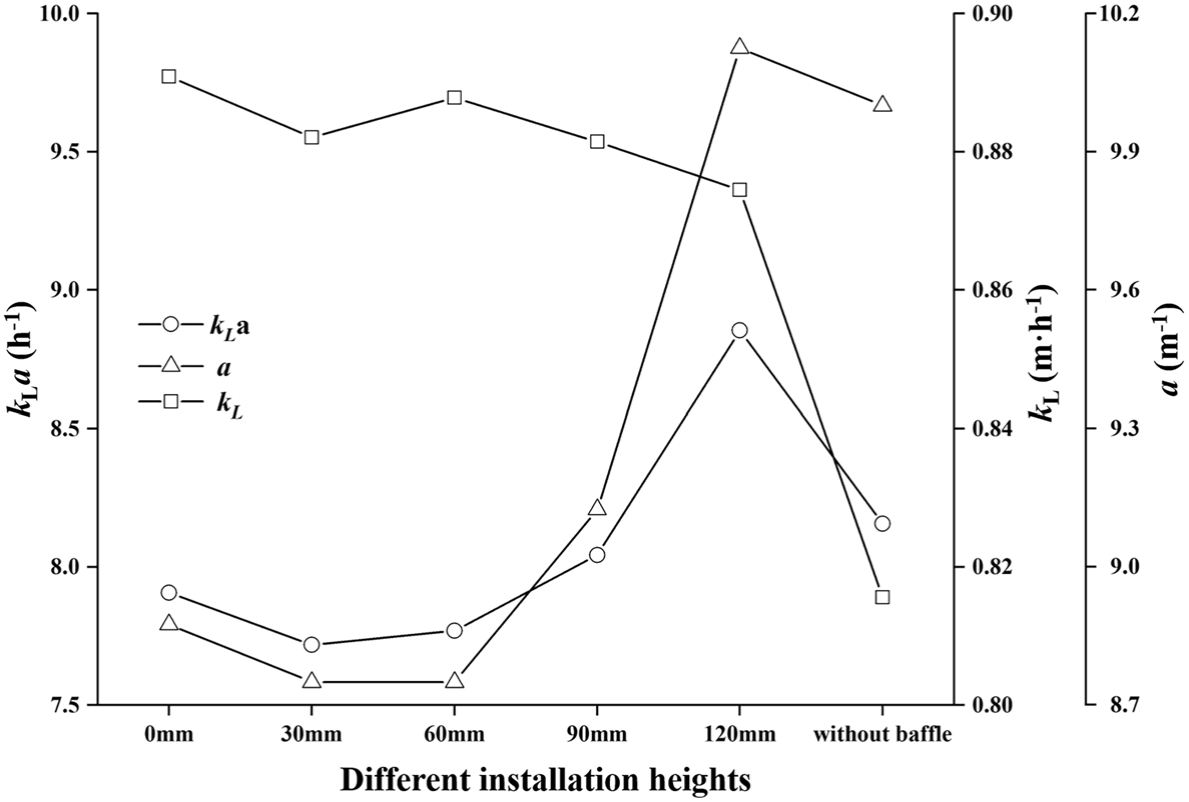
The effect of different installation heights on $k_{L}a$, *A* and $k_{L}a$ in the OSRs at filling volume of 8 L and shaking speed of 100 rpm. The values of $k_{L}$ and *a* were calculated in the hollow OSRs with different installation heights as indicated, separately. The value of $k_{L}a$ was determined based on the result of $k_{L}$ and *a*. The circle ($\bigcirc$ ) represents the value of $k_{L}a$, the triangle ($\triangle$) represents the value of *a*, and the square ($\square$) denotes the value of $k_{L}$.

The volumetric mass transfer coefficient ($k_{L}a$) is crucial parameter for cell cultivation with a high viable cell density. To compare the $k_{L}a$ in the bioreactor with different installation heights, the mass transfer coefficient ($k_{L}$) and the specific interface area (*a*) were simulated at different installation heights. As shown in Fig. 7, the values of $k_{L}$ were nearly a constant for all the studied cases. Under the constant temperature cell culture conditions (37), $k_{L}$ will only depends on the turbulent dissipation rate ($\varepsilon$). Although different installation heights had different effects on the flow field, the value of $\varepsilon$ changed slightly at a fixed shaking speed (see Table 1). Therefore, the values of $k_{L}$ almost did not change at different installation heights.

The values of liquid height ($\Delta h$) were calculated as shown in Table 1. The values of $\Delta h$ remained almost constant at approximately 140 mm, which was slightly higher than the OSR without a hollow baffle, suggesting that the liquid height mainly depended on the shaking speed. Thus, it could be concluded that the hollow baffle had limited influence on the liquid height and even the slope of the wave shape. Due to the constant value of the filling volume, the value of the specific interface area only depends on the interface area. The values of *A* remained almost constant at approximately 0.07 $\mathrm {m^{2}}$ for all the used installation heights (see Table 1), which was probably caused by the Froude number being the same at the fixed shaking speed of 100 rpm. In detail, there was little difference in *A* for different installation heights, and the reason for this could be that the interaction between the hollow wall and liquid wave led to some interface area loss. As shown in Fig. 7, the values of $k_{L}a$ and *a* did not fluctuate greatly. In detail, the values of $k_{L}a$ and *a* had a consistent change trend at different installation heights. When the value of the h$_{\mathrm {L}}$ increased from h$_{\mathrm {L}}$ = 0 mm to h$_{\mathrm {L}}$ = 30mm, the magnitudes of the $k_{L}a$ and *a* decreased slightly. However, the magnitudes of the $k_{L}a$ and *a* increased steadily as h$_{\mathrm {L}}$ increased from h$_{\mathrm {L}}$ = 30 mm to h$_{\mathrm {L}}$ = 120 mm.

### Effect of the hollow cylinder OSR on shear stress

**Figure 8:**
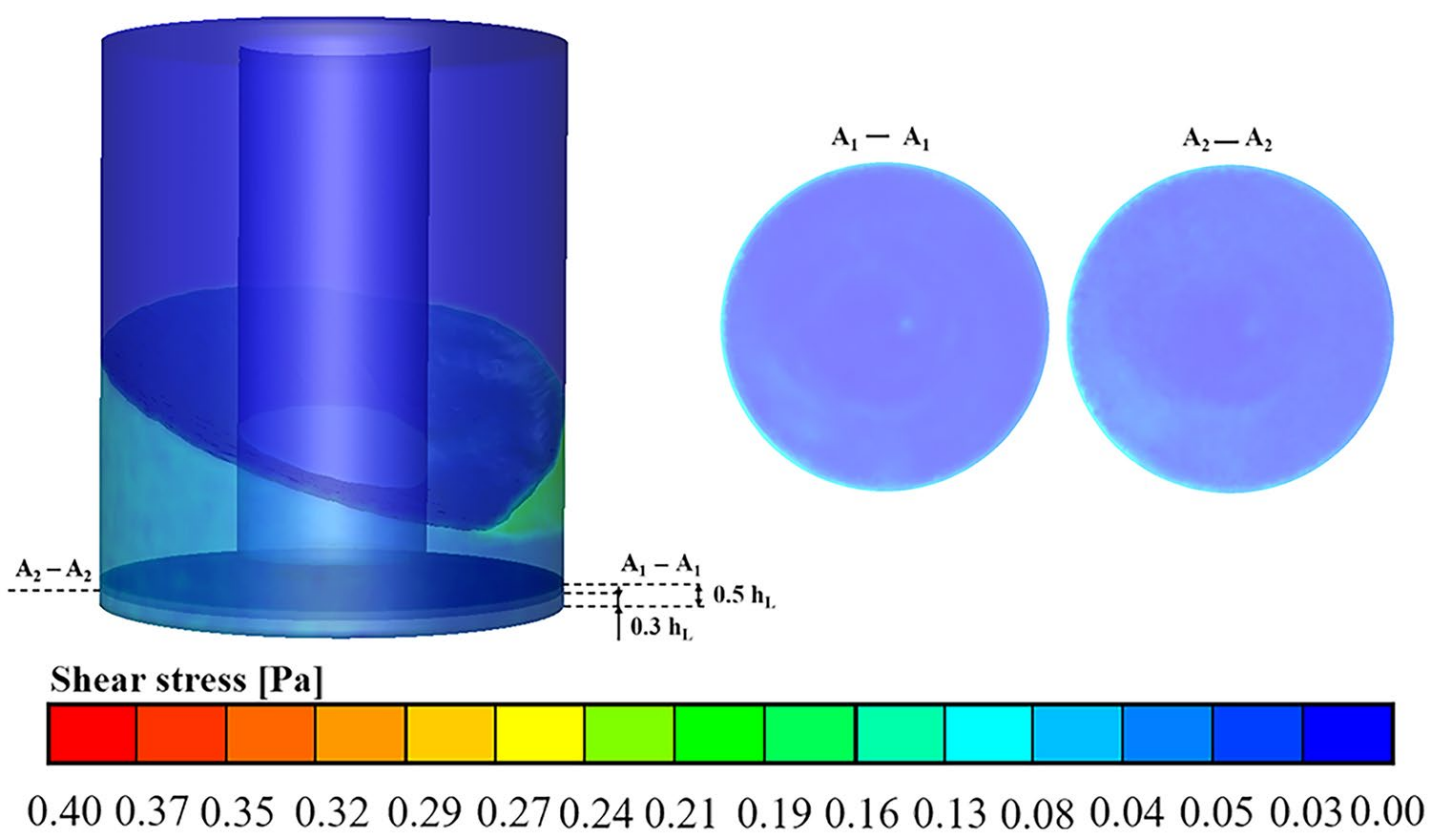
Shear stress distribution of the hollow cylindrical OSR with filling volume of 8 L, shaking speed of 100 rpm and h$_{\mathrm {L}}$ = 30 mm was studied. In order to better visualize the shear stress distribution, the local shear stresses of 0.5 h$_{\mathrm {L}}$ (A$_{1}$-A$_{1}$) and 0.3h$_{\mathrm {L}}$ (A$_{2}$-A$_{2}$) sections were intercepted. The magnitude of the shear stress was indicated by different colors based on the color bars shown in the figure.

Cell damage is a very important problem in bioreactors, but it is normally difficult to analyse^26–29^. Cell damage easily occurs in a fluid environment with large shear stress values^30^. It was reported that a shear stress of 0.4 Pa was a critical value for CHO cells to suffer shear force^31^. To evaluate the hydrodynamic stress environment in the hollow cylindrical OSRs, the shear stress distribution was calculated at a filling volume of 8 L and shaking speed of 100 rpm for the case with h$_{\mathrm {L}}$ = 30 mm (Fig. 8). The reason why the case with an hL of 30 mm was chosen was that the fluid velocity magnitude was higher and the mixing process was intense, as analysed above. By observing the two horizontal Sections A$_{1}$-A$_{1}$ and A$_{2}$-A$_{2}$, the maximum shear stress was located at the outermost edge of the section for both cases. In addition, it could also be seen that the shear stress in horizontal Section A$_{2}$-A$_{2}$ was larger than that in Section A$_{1}$-A$_{1}$. This might be because Section A$_{2}$-A$_{2}$ was closer to the vessel bottom than Section A$_{1}$-A$_{1}$, and the fluid particles would be accelerated more easily near the vessel bottom wall. Meanwhile, the maximum shear stress was found to be approximately 0.2 Pa in the whole vessel, which was lower than the critical value of 0.4 Pa of CHO cells, and large shear stress was located near the wall of the vessel. The results suggested that the fluid environment of the hollow OSR was still gentle for mammalian cell cultivation.

## Conclusion

This study was based on the three-dimensional CFD model, and the reliability of the CFD model was verified by experiments. In this study, we analysed the influence of hollow cylindrical walls on the flow field at a shaking speed of 100 rpm and a filling volume of 8 L. The results showed that the influence of hollow cylindrical walls at different installation heights on the $k_{L}a$ was slight. However,the results showed that the influence of hollow cylindrical walls at different installation heights on the flow field was different. When the installation height was 30 mm, the low-speed zone in the middle region could be greatly reduced, and the mixing of the flow field could also be improved to promote the exchange of matter and energy. At the same time, it was also concluded that the shear stress of the whole flow field was below 0.2 Pa, which was lower than the critical value of 0.4 Pa for CHO cells, indicating that the hollow bioreactor could provide a gentle shear stress environment for mammalian cell cultivation.

## Data Availability

The datasets that pertain to the current study can be made available from the corresponding author on reasonable request.

## Abbreviations

*A*: Interface area ($$\mathrm {m^{2}}$$)
*a*: The specific interface area ($\mathrm {m^{-1}}$)
CFD: Computational fluid dynamics
d: Outer diameter (m)
d$$_{i}$$: Inner diameter (m)
$\varepsilon$: The energy dissipation rate $\left( \mathrm {m^{2} \, s^{-1}}\right)$
$F_{x}$: Centrifugal force in the x direction (N)
$F_{y}$: Centrifugal force in the y direction (N)
$$\mathrm {g}$$: Gravity acceleration ($$\mathrm {m \, s^{-2}}$$)
$$\Delta h$$: Liquid height (m)
h$_{\mathrm {L}}$: Installation height of inter cylinder wall (m)
k$_{L}a$: The volumetric mass transfer coefficient ($\mathrm {h^{-1}}$)
k$_{L}$: The mass transfer coefficient ($\mathrm {m \, h^{-1}}$)
$$l_{0}$$: Characteristic length (m)
N: Shaking speed (rpm)
OSRs: Orbitally shaking bioreactors
*r*: Radius of the hollow cylindrical OSRs (m)
$R_{s}$: Radius of shaking (m)
STRs: Stirred tank bioreactors
*V*: Fluid velocity ($$\mathrm {m \, s^{-1}}$$)
$V_{max}$: Theoretical maximum fluid velocity ($\mathrm {m \, s^{-1}}$)
$V_{L}$: The filling volume (L)
VOF: Volume of fluid model
$\omega$: Angular velocity of shaking ($\mathrm {rad \, s^{-1}}$)
